# Circulating Neutrophils Predict Poor Survival for HCC and Promote HCC Progression Through p53 and STAT3 Signaling Pathway

**DOI:** 10.7150/jca.42953

**Published:** 2020-04-06

**Authors:** Yan Wang, Rongrong Yao, Danying Zhang, Rongxin Chen, Zhenggang Ren, Lan Zhang

**Affiliations:** 1 Department of Hepatic Oncology, Zhongshan Hospital, Fudan University, Shanghai 200032, P. R. China; 2 Department of Oncology, Huashan Hospital, Fudan University, Shanghai 201907, P.R.China; 3 Department of Gastroenterology, Zhongshan Hospital, Fudan University, Shanghai 200032, P. R. China

**Keywords:** HCC, neutrophils, survival, p53, STAT3

## Abstract

**Background**: Tumor-associated neutrophils (TANs) contribute to tumor progression, invasion, and angiogenesis. However, most studies focus on tumor infiltration neutrophils while the roles of circulating neutrophils in tumor progression remain unclear. This study was aimed to verify the pro-tumor effects of circulating neutrophils and its' mechanism in HCC.

**Methods**: We collected clinical data of 127 HCC patients underwent TACE. The prognostic factors for overall survival (OS) were analyzed by Kaplan-Meier curve and Cox models. Circulating neutrophils of HCC patients were sorted and co-cultured with human HCC cell lines MHCC-97H and SMMC-7721. Then we detected tumor cells' proliferation, migration, and invasion. Phosphokinase array was used to determine the kinase profile on MHCC-97H and SMMC-7721 cultured with or without circulating neutrophils.

**Results**: The result of multivariate analyses of 127 patients showed that increased circulating neutrophils was an independent poor prognostic factor for OS of HCC patients underwent TACE. Circulating neutrophils promoted migration and invasion of HCC cell lines but had no impact on proliferation. The kinase profile on HCC cell lines showed that p-p53^S46^ and p-STAT3^Y705^ were up-regulated after co-cultured with circulating neutrophils. Repeated scratch tests and transwell tests showed a reversed impact on migration and invasion of circulating neutrophils after we treated HCC cell lines with inhibitors of p53 or STAT3.

**Conclusion**: Circulating neutrophils was an independent poor prognostic factor for OS of HCC patients underwent TACE. It had pro-tumor effect on HCC through p53 and STAT3 signaling pathway.

## Introduction

Neutrophil is a substantial proportion of the tumor microenvironment in a wide variety of cancer types. Tumor-associated neutrophils (TANs) has been reported to have pro-tumor effect in tumor progression, angiogenesis, invasion and immune suppression 1, 2. In hepatocellular carcinoma (HCC), high intratumoral or peritumoral neutrophils are correlated with overall survival (OS) 3, 4. TANs recruit macrophages and Treg cells to HCC to promote their growth, progression, and resistance to sorafenib 5. Tumor microenvironment induces impaired antitumor immunity via the modulation of PD-L1 expression on tumor infiltrating neutrophils in HCC 6. Since most HCC patients were diagnosed at late stage and tumors were unresectable, it was difficult to detected TANs and its changes in tumor microenvironment. Currently more and more researches began to focus on circulating neutrophils. However, the function and mechanism of circulating neutrophils in cancer have only begun to be investigated over the past decades. This study was aimed to verify the pro-tumor effects of circulating neutrophils and its' mechanism in HCC.

## Patients and Methods

### Patients and Follow-Up Evaluation

Clinical data of HCC patients underwent TACE from November 2011 to December 2012 at Department of Hepatic Oncology, Zhongshan Hospital (Fudan University, China) were collected with informed consent form signed off. 127 HCC patients were enrolled in the study. HCC were diagnosed based on pathology or clinical diagnostic criteria by AASLD. Exclusion criteria are 1) Received any anticancer therapy before TACE 2) Received any therapy by colony stimulating factors within six months before enrollment. Follow-up tests included ultrasound, AFP measurements (every 2‐3 months), and contrast‐enhanced CT or MRI (every 6 months). The last follow‐up was in June 2015. The study was approved by the research ethics committee of Zhongshan Hospital.

### Neutrophils Isolation

For neutrophils isolation, peripheral blood samples were collected and added into a human circulating neutrophil separating kit (Tianjin Hao Yang Biotechnology Co, Ltd. China) within two hours. Density gradient centrifugation was performed according to the manufacturer's protocol. We confirmed purification of neutrophils via fluorescence-activated cell sorter analysis with anti-CD66b antibody (BD Pharmingen, USA) and Wright's staining, showing a purity of greater than 95%.

### Cell Lines and Cell Culture

Highly metastatic human HCC cell line MHCC- 97H was established in the Liver Cancer Institute, Fudan University (Shanghai, China). HCC cell line SMMC-7721 was obtained from the Cell Bank of Shanghai Institutes of Biological Sciences, Chinese Academy of Sciences. As for co-cultured groups, cell lines were cultured in RPMI.1640 supplemented with 20% fetal bovine serum (FBS, Gibco BRL, USA), circulating neutrophils were added into culture medium at the proportion of 1:1 (cell counts) after 24 hours. All cells were incubated at 37°C in a humidified atmosphere containing 5% CO_2_.

### Cell Proliferation, Migration and Invasion Assays

To detect cell proliferation, cells were plated at a density of 5,000 cells/well in triplicate in 96-well culture plates. The OD value at 450 nm was measured at 1day, 2days, 3days, 4days and 5days after cultured with or without neutrophils via CCK8 kit (Beyotime, Shanghai). Wound-healing assays were performed after 48hours cultured with or without neutrophils (3 x 10^5^cells/well, 6-well culture plates) to detect cell migration. Cell invasion assays were performed in 24-well transwells with an 8.0 μm pore polycarbonate membrane insert (Corning, USA). In total, 3 × 10^4^ HCC cells were suspended in 200 μL PRIM.1640 and added to the upper chamber with Matrigel-coated filters. The lower chamber was filled with 800 μL PRIM.1640 with 20% FBS (Gibco BRL, USA) with or without 6× 10^4^ cells of neutrophils. After incubation for 24hours, the cells on the upper surface of the membrane were removed and the migrated cells on the lower surface were fixed in 4% paraformaldehyde, stained with 0.1% crystal violet for 15 minutes at room temperature and counted (10 fields) under a ×100 objective. The mean ± standard deviation (SD) was then calculated. In the inhibitor groups, the concentration of inhibitor of STAT3 (HO-3867, MCE, USA)was 15μmol/L and the concentration of inhibitor of p53 (Pifithrin-β, MCE,USA) was 3µmol/L. All experiments were performed three times.

### Phosphokinase Array Analysis

HCC cells were sorted 48hours after cultured with or without neutrophils. The phosphokinase array was performed using the human phosphokinase array blot to detect the relative levels of phosphorylation of 43 kinase phosphorylation sites and 2 related total proteins (catalogue number: ARY003; R&D Systems). Protein lysate was incubated with the array membrane and protein signal was visualized using a chemifluorescence detection system (Bio-Rad) according to the manufacturer's protocol. The relative intensity of specific protein expression was determined using Quantity One (Bio-Rad, Hercules, CA) software 5.

### Statistical Analyses

Statistical analyses were performed using SPSS 19.0 for Windows (SPSS, Chicago, IL). All consecutive data were expressed as mean ± standard deviation. Comparison of qualitative variables was performed by the *x*^2^ test or Fisher's exact test. OS was defined as the interval between the date of the first TACE treatment and endpoint event (death or censored on the last follow-up). OS were calculated by the Kaplan-Meier curve and the differences of survival between subgroups were compared by the log-rank test. Prognostic variables on univariate analysis were entered into subsequent multivariate analysis using Cox's proportional hazard model. P<0.05 was considered statistically significant.

## Results

### Increased circulating neutrophils was an independent poor prognostic factor for OS of HCC underwent TACE

**Figure 1:**
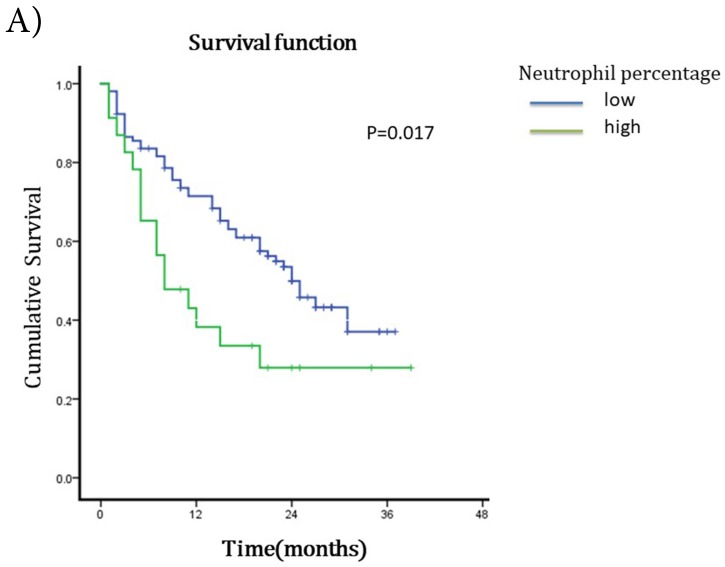

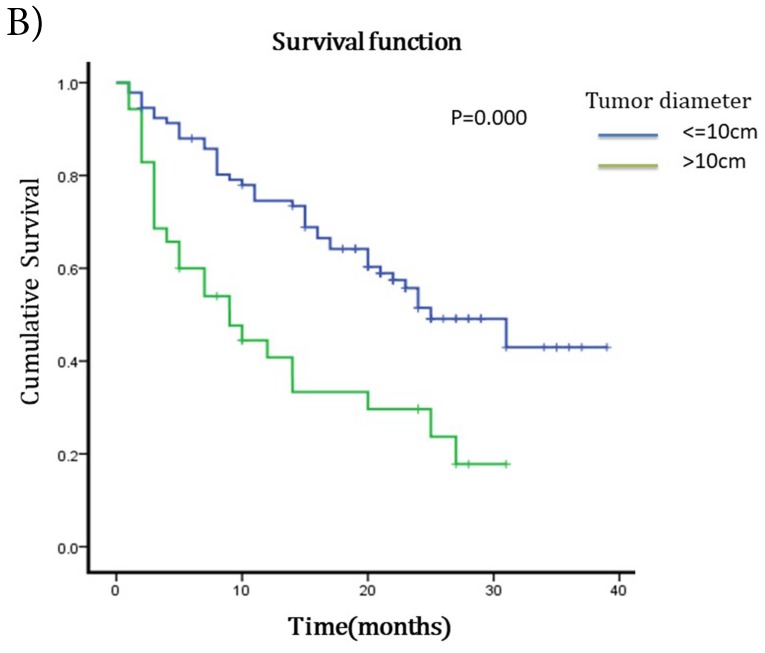

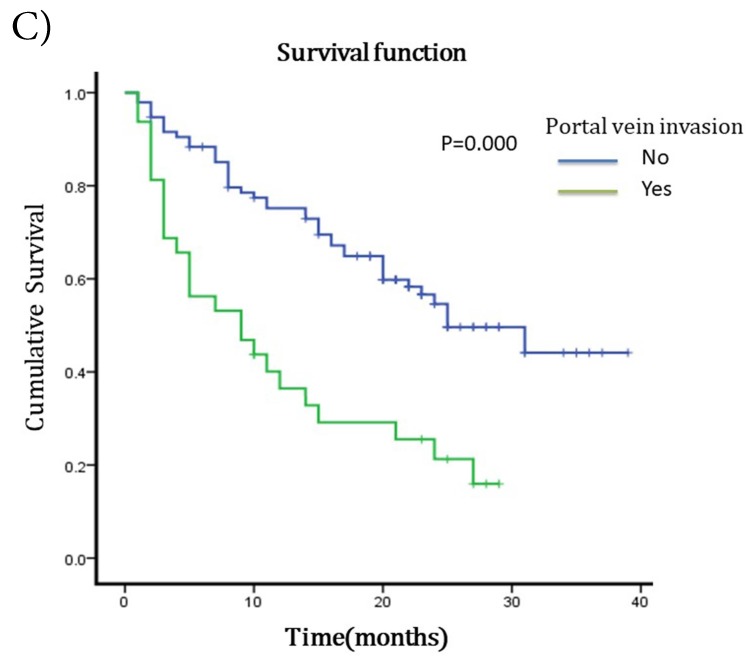
** Survival curves for different groups of HCC patients underwent TACE. A)** Survival curves for high circulating neutrophils group (>70%) and low circulating neutrophils group (<=70%). OS for high circulating neutrophils group was 16.25 months while 22.92 months for low group(p=0.017,HR=1.9, 95%CI:1.086-3.325). **B)** Survival curves for different tumor diameter groups. OS for tumor diameter >10cm group was 13.25 months while 25.22 months for tumor diameter <=10cm group (p=0.000, HR=1.812, 95%CI:1.027-3.197). **C)** Survival curves for groups with or without portal vein invasion. OS for portal vein invasion group was 12.12 months while 25.42 months for without portal vein invasion group (p=0.000, HR=1.930, 95%CI:1.104-3.373).

The median survival after TACE was 23 months (95%CI 17.51~28.49). The 1-year and 2-years survival rates were 60.63% and 19.69%. To understand the prognostic factors for OS, we analyzed clinic pathological parameters of HCC patients underwent TACE by Kaplan-Meier curve. The cut-off value of circulating neutrophil percentage was 70% (the normal upper limit of peripheral blood in healthy people). Univariate analysis showed that high circulating neutrophil percentage, γ-glutamyltransferase (GGT), pretreatment serum AFP status, ascites, tumor diameter, portal vein invasion and metastasis were potential influence factors for OS (Table 1). Multivariate analysis by Cox's proportional hazard model showed that high circulating neutrophil percentage (Table 1, Fig 1A), in addition to tumor diameter more than 10cm (Table 1, Fig 1B) and portal vein invasion (Table 1, Fig 1C), was an independent poor prognostic factor for OS. The OS for HCC patients in high circulating neutrophil percentage group was shorter than that in low group (16.25 months vs 22.92 months, p=0.017, HR=1.9).

There was no difference in demographics, serum biochemistries or tumor-related characteristics between low neutrophil percentage group and high neutrophil percentage group (Table 2).

### Circulating neutrophils promoted migration and invasion of HCC cell lines, but had no impact on cell proliferation

**Figure 2:**
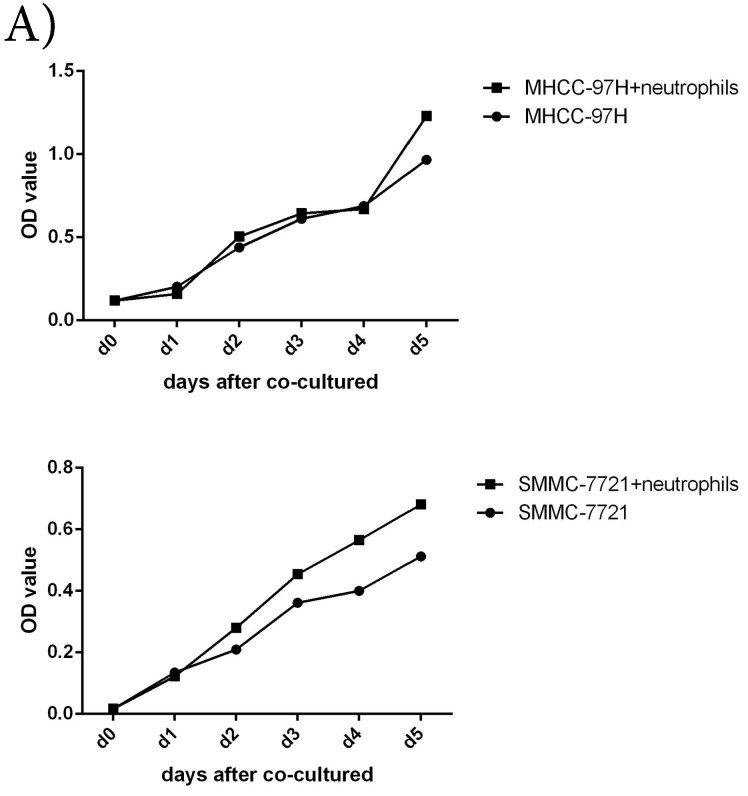

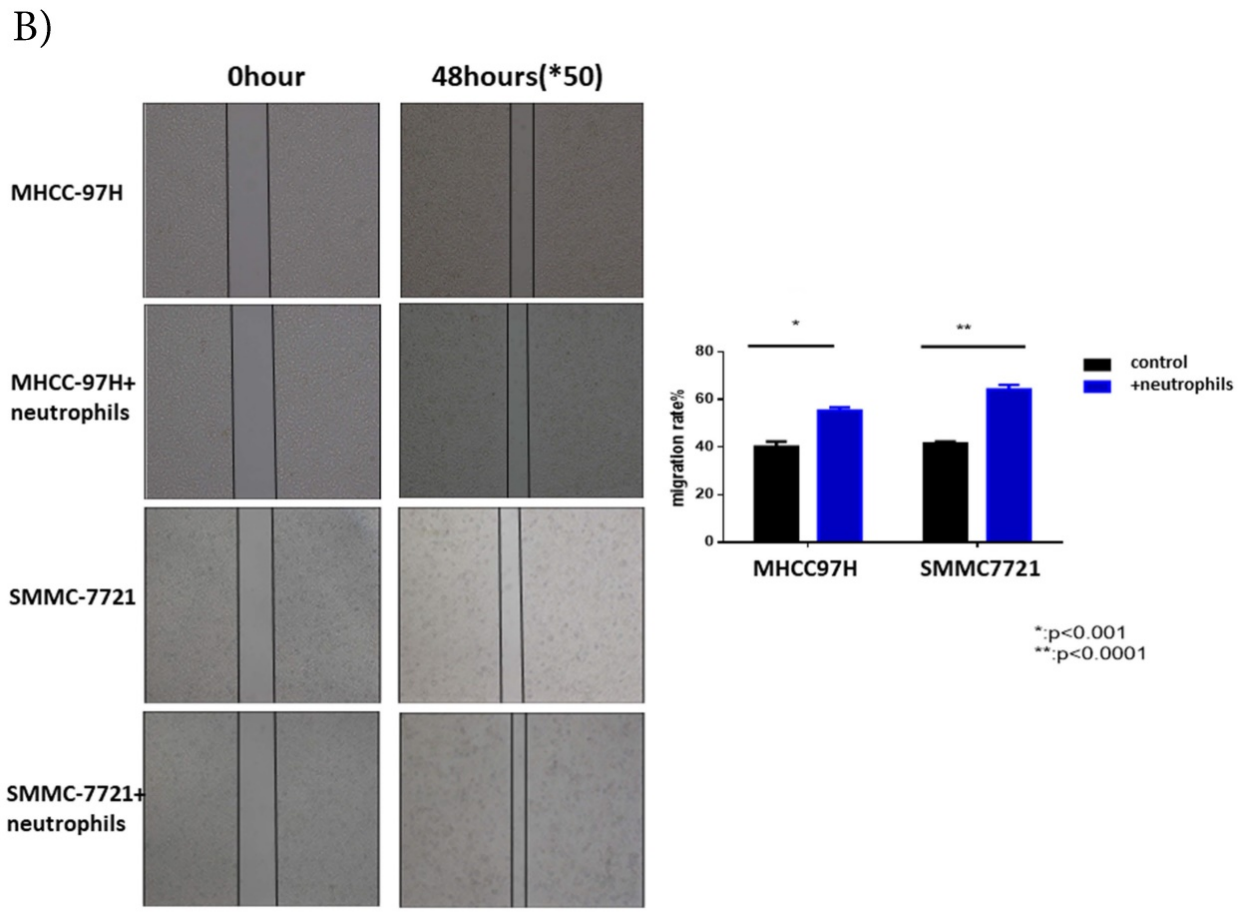

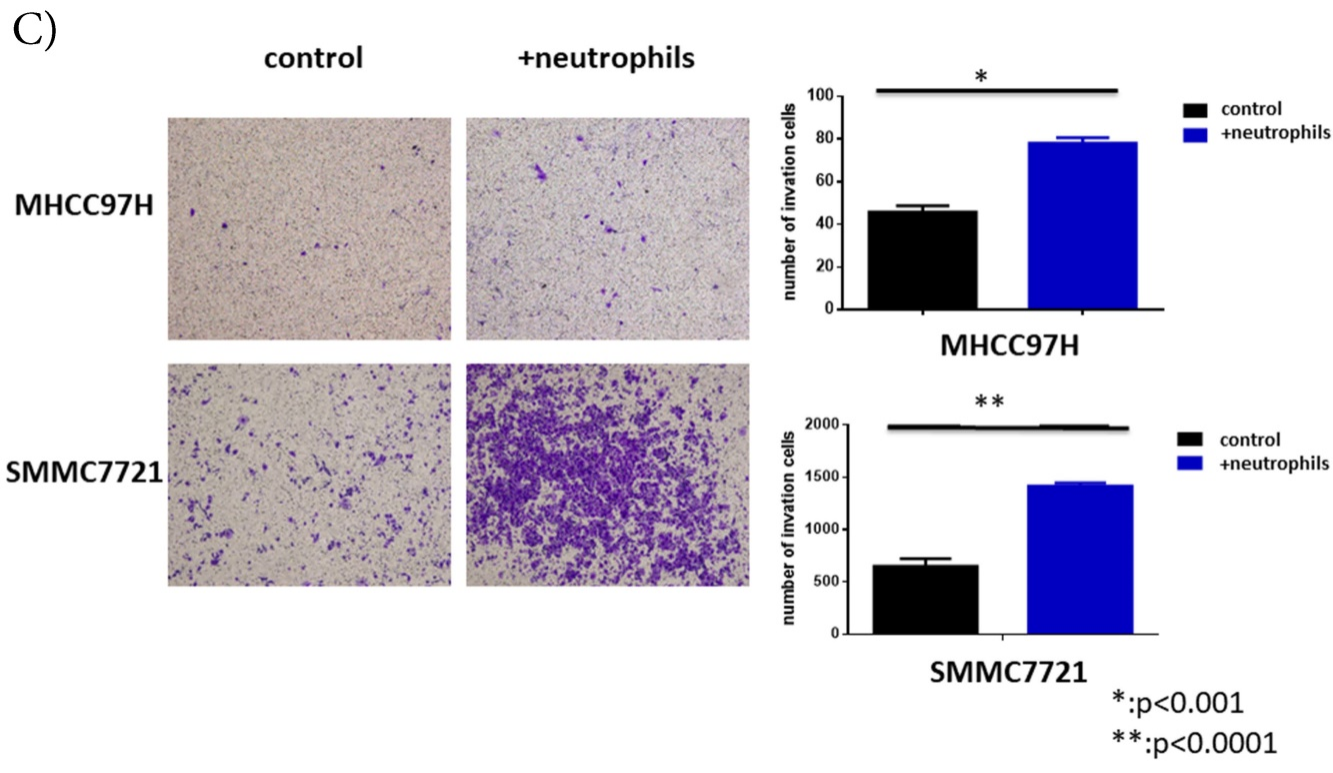
** The functions of circulating neutrophils in HCC. A)** Cell proliferation of HCC cell lines MHCC-97H and SMMC-7721 after 1day,2days,3days,4days and 5days cultured with or without circulating neutrophils were detected via CCK8 kit. There's no difference on cell proliferation between two groups. **B)** Cell migration of HCC cell lines MHCC-97H and SMMC-7721 after cultured with or without circulating neutrophils were detected by Wound-healing assays. After 48hours cultured with circulating neutrophils, the cell migration rates of MHCC-97H and SMMC-7721 were increased than control groups. **C)** Cell invasion assays of HCC cell lines MHCC-97H and SMMC-7721 were performed after 24hours incubation with or without circulating neutrophils, cells in purple were invasion cells. The number of invasion cells of co-cultured groups were increased than control groups.

To further understand the functions of circulating neutrophils in HCC, we isolated neutrophils from periphery blood cells of HCC patients. Cell proliferation, migration and invasion of HCC cell lines MHCC-97H and SMMC-7721 were detected after cultured with or without circulating neutrophils. There was no difference on cell proliferation between two groups (Fig 2A). But migration and invasion of HCC cell lines both increased after co-cultured with circulating neutrophils (Fig 2B, Fig 2C). It showed circulating neutrophils promoted migration and invasion of HCC cell lines, but had no impact on cell proliferation.

### p-p53 and p-STAT3 were up-regulated in HCC after co-cultured with circulating neutrophils, while p53 and STAT3 inhibitors reversed the effect on migration and invasion

**Figure 3:**
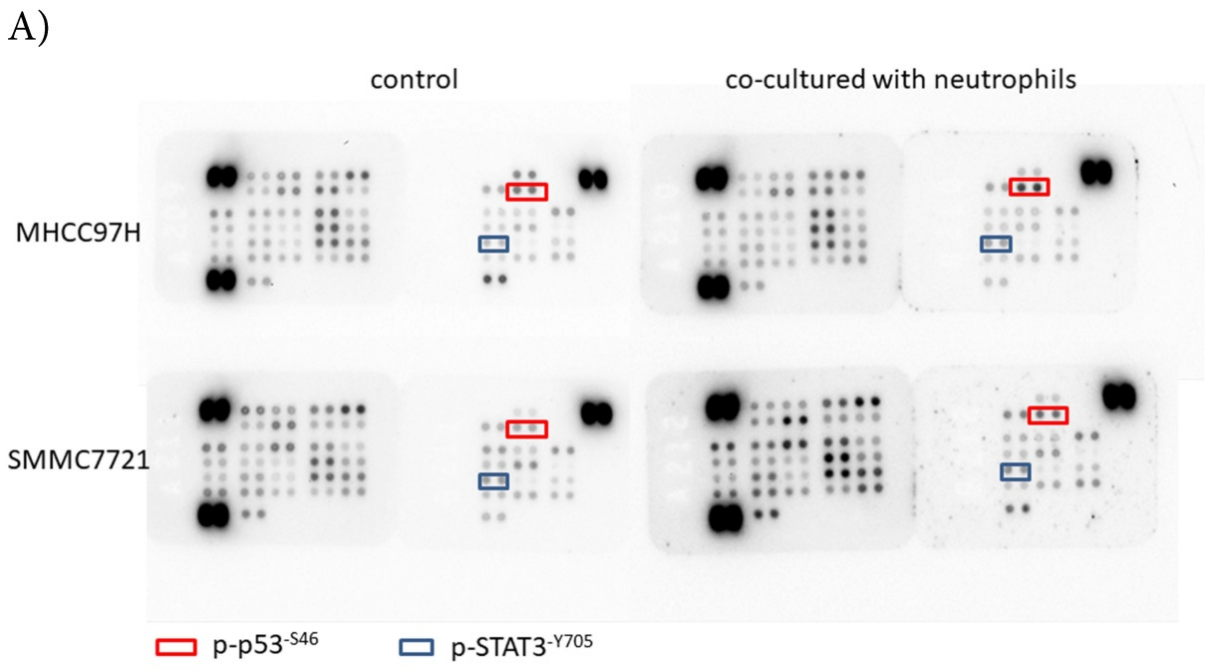

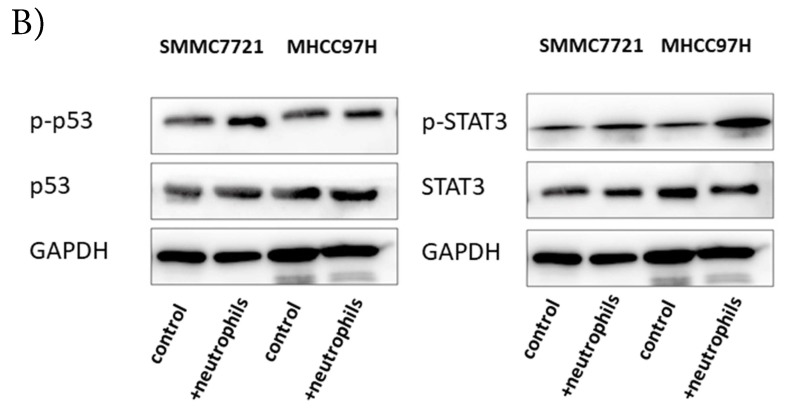

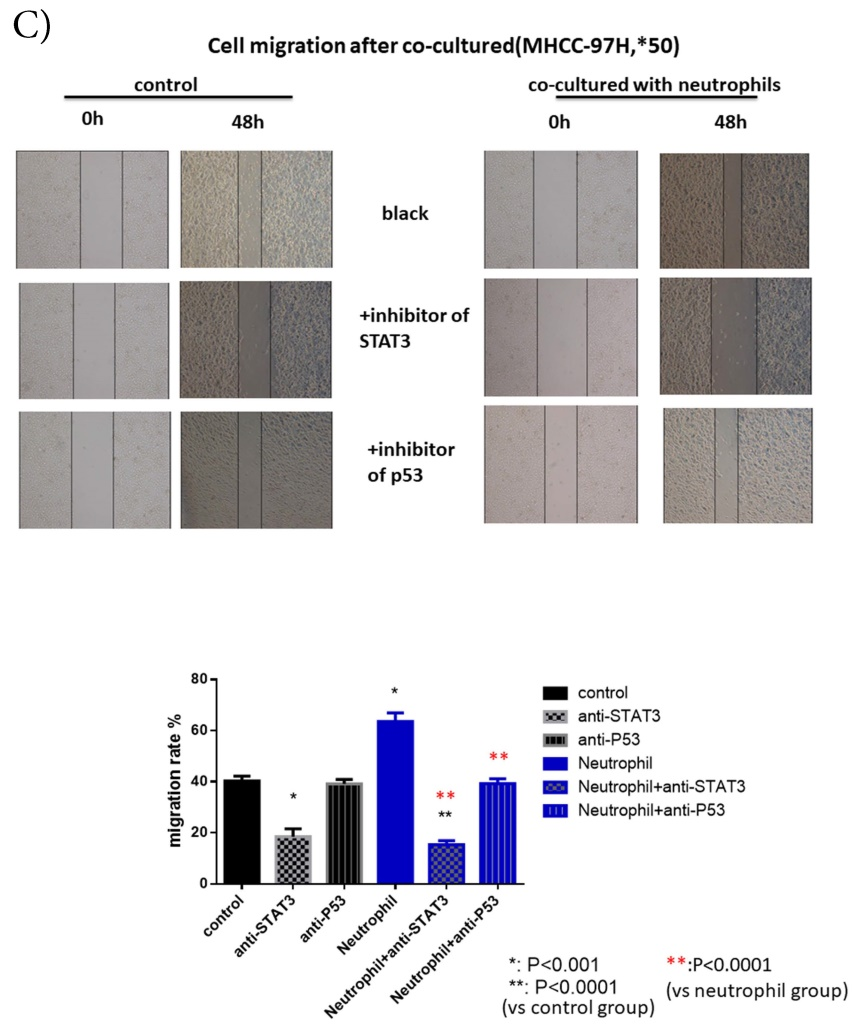

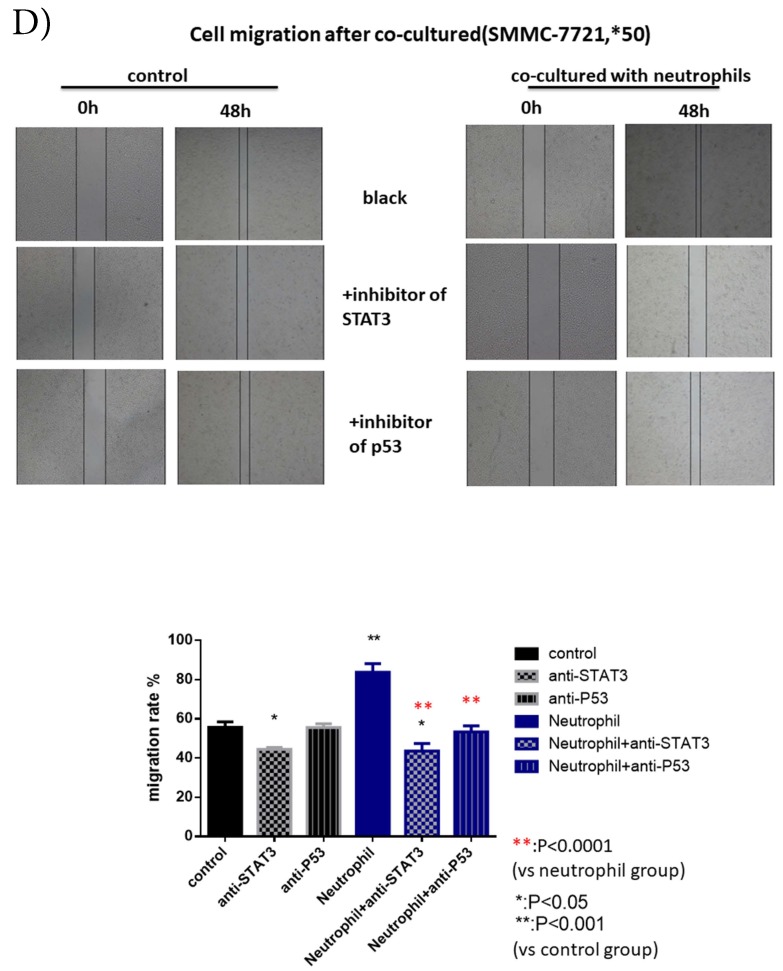

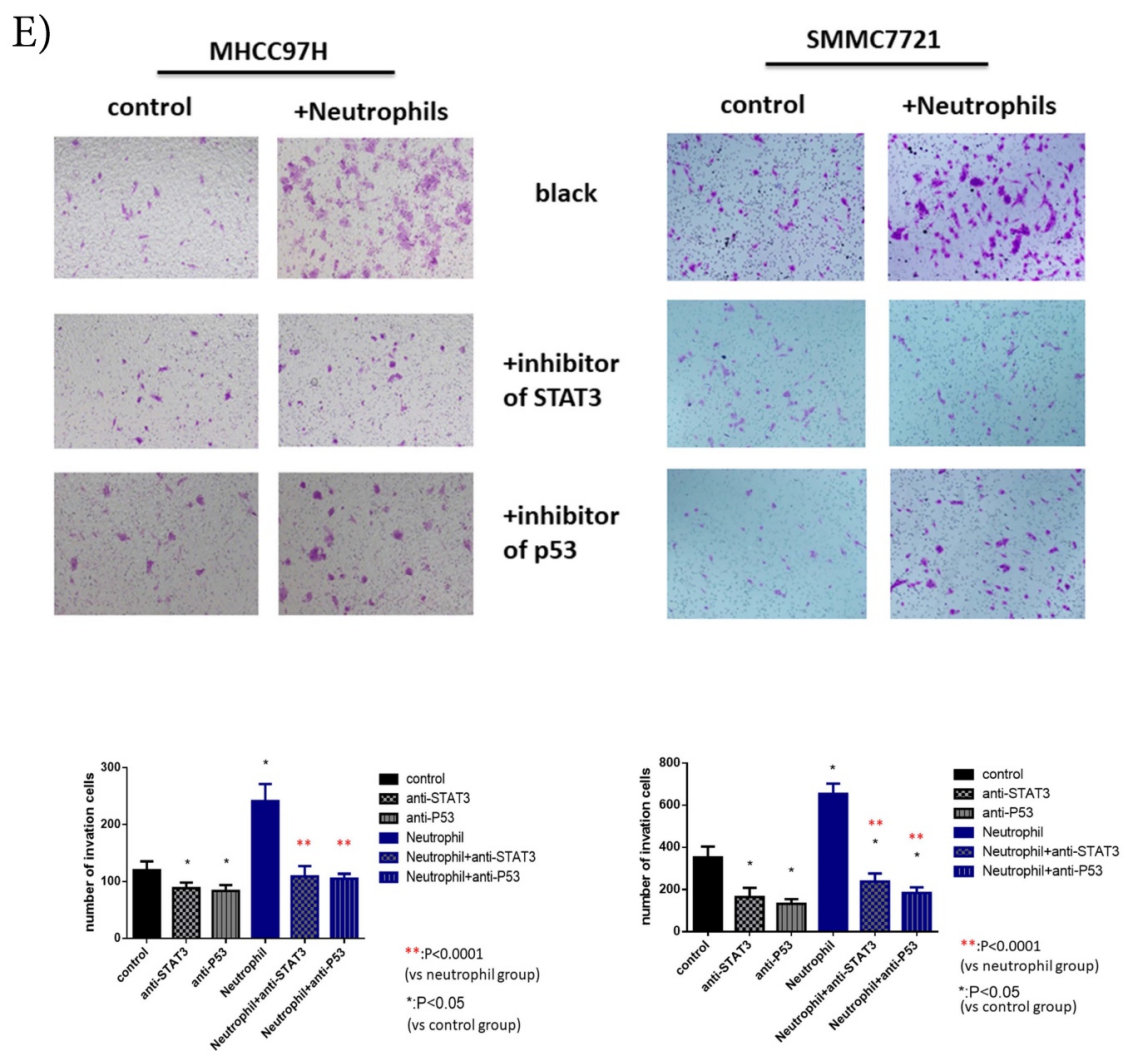
** p-p53 and p-STAT3 were up-regulated in HCC after co-cultured with circulating neutrophils while inhibitors of p53 and STAT3 reversed the effect on migration and invasion. A)** Phosphokinase arrays on HCC cell lines cultured with or without circulating neutrophils were detected and p-p53^S46^ and p-STAT3^Y705^ were up-regulated after co-cultured with circulating neutrophils both in MHCC-97H and SMMC-7721 cell lines. **B)** p-p53 and p-STAT3 were increased in MHCC-97H and SMMC-7721 after co-cultured with circulating neutrophils by Western Blot. **C and D)** Cell migration rates of MHCC-97H (C) and SMMC-7721 (D) increased after co-cultured with circulating neutrophils, but decreased when treated with inhibitors of p53(Pifithrin-β) and STAT3(HO-3867). **E)** Number of invasion cells of MHCC-97H and SMMC-7721 increased after co-cultured with circulating neutrophils, but decreased when treated with inhibitors of p53(Pifithrin-β) and STAT3(HO-3867) (cells in purple were invasion cells).

To explore the mechanism by which circulating neutrophils educated HCC cells to become more progressive, we analyzed a phosphokinase array on human HCC cell lines cultured with or without circulating neutrophils to determine the kinase profile of several distinct signaling pathways. We found that p-p53^S46^ and p-STAT3^Y705^ were both up-regulated in two HCC cell lines in response to co-culture with circulating neutrophils (Fig 3A). These data were validated by Western blot (Fig 3B).

After we treated HCC cell lines with inhibitors of p53 (Pifithrin-β) and STAT3(HO-3867), we found a respective and complete reversion of the increased migration and invasion of HCC cell lines by circulating neutrophils (Fig 3C, Fig 3D, Fig 3E). These results suggested that the promotion effect on HCC cell lines by circulating neutrophils required p53 and STAT3 signaling pathways.

## Discussion

A further understanding of the interactions between immune cells and cancer cells will bring new breakthrough in cancer therapies. Previous focus was often on the interactions in the primary tumor sites and its microenvironment while the role of immune cells during cancer dissemination in patients remains uncharacterized.

Although TANs are reported to have pro-tumor effect in tumor progression, angiogenesis, invasion and immune suppression, the roles of circulating neutrophils remain unclear. Prof Stefanie K. Wculek 7 identified neutrophils as the main component and driver of metastatic establishment within the (pre-) metastatic lung microenvironment in a mouse breast cancer model. He demonstrated that neutrophils specifically support metastatic initiation. He also found that neutrophil-derived leukotrienes aid the colonization of distant tissues by selectively expanding the sub-pool of cancer cells that retain high tumorigenic potential. Barbara Maria Szczerba reported that neutrophils directly interact with CTCs to support cell cycle progression in circulation and to accelerate metastasis seeding in breast cancer 8. Neutrophil- to-lymphocyte ratio (NLR) has been introduced as a prognostic factor for survival in many tumor types including HCC 9. However, in most high risk HCC areas (China), the key determinants are chronic HBV infection 10, the NLR can't fully represent the effect of neutrophils because it might be influenced by changes of lymphocyte count caused by liver cirrhosis. In our study, we chose circulating neutrophils as target cell to represent the effect of circulating neutrophils directly. Our results showed that circulating neutrophils was an independent poor prognostic factor for OS of HCC patients underwent TACE. Circulating neutrophils promoted migration and invasion of HCC which confirmed its pro-tumor effects like TANs in HCC.

Neutrophils can influence tumor development by modulating the recruitment, profile and phenotype of other immune cells 1. In this study, we focused on the interactions between circulating neutrophils and tumor cells. We demonstrated that circulating neutrophils played a direct role in promoting HCC progression through p53 and STAT3 signaling pathway. p53 plays multifunctional roles in different stages in HCC^11^. Alterations in p53 signaling pathways are considered as pro-oncogenic by acting on cell proliferation, survival, invasion and immune evasion 12. STAT3 is phosphorylated in 60% of human HCC and active STAT3 correlates with tumor aggressiveness 13. The Jak/STAT signaling cascade is a central signaling hub that can be activated by a plethora of cytokines, growth factors and hormones, one important of them is the interleukin-6 (IL-6) family of cytokines 14, 15. We supposed that circulating neutrophils active STAT3 signaling pathway by priming IL-6 but further researches were needed. Possible strategies for targeting the neutrophils/IL-6/STAT3 pathway or certain extracellular cytokines in HCC therapy may be achieved in the future.

## Figures and Tables

**Table 1:** Univariate and multivariate analyses of prognostic factors for overall survival of HCC underwent TACE

	N (n=127)	OS (months)	Univariate	Multivariate	
			p value	HR(95%CI)	p value
**gender**					
male	107	22.85	0.329		NA
female	20	18.21			
**age**					
>65y	86	21.99	0.753		NA
<65y	41	21.89			
**Total bilirubin(μmol/l)**					
<=17.1	109	22.57	0.599		NA
>17.1	18	19.44			
**Albumin(g/l)**					
>=35	104	21.97	0.606		NA
<35	23	21.14			
**GGT(U/L)**					
<=70	58	26.09	0.004*	1.178 (0.657~2.112)	0.582
>70	69	18.63			
**Prothrombin time(s)**					
<=14	106	21.11	0.068		NA
>14	21	26.46			
**Hemoglobin(g/l)**					
<=110	10	15.90	0.206		NA
>110	117	22.79			
**Neutrophil percentage (%)**					
>70%	23	16.25	0.017*	1.900 (1.086~3.325)	0.025*
<=70%	104	22.92			
**platelet(10^9/L)**					
>=100	95	21.70	0.370		NA
<100	32	23.17			
**HbsAg**					
positive	100	23.16	0.182		NA
negative	27	18.16			
**HCV-Ab**					
positive	6	15.67	0.937		NA
negative	121	22.38			
**pretreatment AFP(ng/ml)**					
>20	75	18.94	0.003*	1.514 (0.883~2.596)	0.131
<=20	52	25.16			
**ascite**					
yes	12	9.47	0.002*	1.923 (0.921~4.015)	0.082
no	115	23.42			
**numbers of tumor**					
single	69	21.67	0.727		NA
mutiple	58	22.23			
**Tumor diameter(cm)**					
>10	35	13.25	0.000*	1.812 (1.027~3.197)	0.040*
<=10	92	25.22			
**portal vein invasion**					
yes	32	12.12	0.000*	1.930 (1.104~3.373)	0.021*
no	95	25.42			
**metastasis**					
yes	20	13.2	0.001*	1.927 (0.988~3.757)	0.054
no	107	23.95			

*p<0.05

**Table 2:** Clinic pathological characteristics for patients between low circulating neutrophil group and high circulating neutrophil group

		Low circulating neutrophil (<=70%) (n=104)	High circulating neutrophil (>70%) (n=23)	p value
**gender**	male	86	21	0.526
	female	18	2	
**age**	>65y	32	9	0.466
	<=65y	72	14	
**Total bilirubin (μmol/l)**	<=17.1	91	18	0.319
	>17.1	13	5	
**Albumin (g/l)**	>=35	88	16	0.131
	<35	16	7	
**GGT (U/L)**	<=70	48	10	0.501
	>70	56	13	
**Prothrombin time(s)**	<=14	87	19	0.557
	>14	17	4	
**HbsAg**	positive	84	16	0.263
	negative	20	7	
**HCV-Ab**	positive	4	2	0.297
	negative	100	21	
**Pretreatment AFP (ng/ml)**	>20	61	14	0.297
	<=20	43	9	
**ascite**	yes	7	5	0.042
	no	97	18	
**numbers**	single	59	10	0.259
	mutiple	45	13	
**diameter (cm)**	>10	30	5	0.610
	<=10	74	18	
**portal vein invasion**	yes	26	6	0.551
	no	78	17	
**metastasis**	yes	15	5	0.360
	no	89	18	
**Child-pugh stage**	A	95	20	0.454
	B	9	3	
**BCLC stage**	A+B	72	16	0.975
	C	32	7	
